# Preventing the Silent Threat: A Perspective on Preparing Bangladesh for Human Metapneumovirus (HMPV)

**DOI:** 10.1002/hsr2.71101

**Published:** 2025-08-27

**Authors:** Md. Jubayer Hossain, Soniya Akter Sony, Fatema Tuj Johora Fariha, Sajjad Hossen

**Affiliations:** ^1^ Center for Health Innovation, Research, Action, and Learning—Bangladesh (CHIRAL Bangladesh) Dhaka Bangladesh; ^2^ Population Health Studies Division Center for Health Innovation, Research, Action, and Learning—Bangladesh (CHIRAL Bangladesh) Dhaka Bangladesh; ^3^ Big Bioinformatics Lab Center for Health Innovation, Research, Action, and Learning—Bangladesh (CHIRAL Bangladesh) Dhaka Bangladesh

**Keywords:** Bangladesh, health policy and planning, HMPV diagnostics and treatment, human metapneumovirus (HMPV), public health preparedness, respiratory virus surveillance

## Abstract

**Background and Aims:**

Human metapneumovirus (HMPV) is a significant respiratory pathogen with a global presence that disproportionately affects young children and the elderly. In Bangladesh, HMPV remains under‐recognized, despite its contribution to respiratory morbidity among children. This Perspective underscores the importance of HMPV as an emerging public health concern in Bangladesh, and advocates for the enhancement of national preparedness and response strategies.

**Methods:**

This article synthesizes existing evidence from the published literature and surveillance reports to evaluate the epidemiological profile of HMPV, its public health implications, and the capacity of the Bangladeshi health system to respond. The focus is on identifying systemic challenges and potential policy responses.

**Results:**

The analysis suggests that the actual burden of HMPV in Bangladesh is likely underestimated owing to limited surveillance, inadequate diagnostic infrastructure, and low public awareness. The country's health system encounters challenges including insufficient funding for research, scarcity of laboratory resources, and competing health priorities. Despite these obstacles, there are opportunities to enhance HMPV preparedness through the development of national surveillance systems, capacity building, and strategic partnerships with global health organizations.

**Conclusion:**

A comprehensive strategy is essential to effectively address the threats posed by HMPV in Bangladesh. This strategy should encompass the development of targeted surveillance systems, implementation of public awareness initiatives, enhancement of diagnostic capabilities, and fostering of international collaboration. By investing in these areas, Bangladesh can improve its ability to detect, prevent, and manage HMPV outbreaks, thereby reducing the overall burden of respiratory disease.

## 1 Background

Emerging infectious diseases pose significant challenges to global health, especially in resource‐limited settings where healthcare systems are often underprepared for new threats [1, 2, 3]. Among the respiratory viruses, human metapneumovirus (HMPV) is a notable pathogen that affects individuals globally. While not a new virus, its clinical burden remains underrecognized in many countries, including Bangladesh. However, improved surveillance for respiratory viruses overall—such as HMPV, influenza, and respiratory syncytial virus (RSV)—is vital to prepare for potential future epidemics [4, 5]. The virus is classified under the *Pneumoviridae family* and genus Metapneumovirus. HMPV infections affect individuals of all age groups, with severe cases occurring primarily in children under 2 years of age and older adults [6, 7, 8]. Since its initial identification in 2001 [8], HMPV has been reported in every continent, with its prevalence varying significantly [9]. Almost all children are infected with HMPV by the age of five [8, 10].

The clinical manifestations of HMPV infection range from mild upper respiratory tract symptoms to severe lower respiratory tract disease [11], often resembling those caused by other common respiratory viruses, particularly RSV [12, 13]. HMPV is primarily diagnosed via reverse‐transcription polymerase chain reaction (RT‐PCR) because culturing the virus is challenging [14]. Infection does not provide lasting immunity, leading to reinfection throughout life [15]. Despite its considerable impact on public health, there are no approved vaccines or antiviral treatments for HMPV to date, although ongoing research holds promise for future therapeutic options [16]. The virus has a substantial clinical and socioeconomic impact, contributing to increased healthcare utilization and missed workdays during outbreaks [17].

Preventive measures are crucial to mitigate the impact of HMPV. These include enhancing surveillance systems for early detection, improving diagnostic capabilities, and implementing public health campaigns to educate the population about the virus and its transmission [18, 19]. Additionally, the development of effective vaccines and antiviral therapies is essential to protect at‐risk populations and reduce the incidence of severe diseases [15, 20].

In late 2024, China experienced a significant increase in HMPV infections, particularly among children under the age of 14 [21]. From December 16 to 22, HMPV accounted for 6.2% of positive tests for respiratory illnesses and 5.4% of respiratory‐related hospitalizations [22]. These rates surpassed those of COVID‐19, rhinovirus, and adenovirus during the same period [23]. By early January 2025, India reported its first confirmed cases of HMPV. The Indian Council of Medical Research (ICMR) confirmed seven cases, mainly affecting infants and toddlers [24]. These cases were identified in the states of Karnataka and Gujarat, emphasizing the potential regional threats posed by the HMPV.

The prevalence and impact of HMPVs among children in urban areas have been studied in Bangladesh. In addition, a nationwide surveillance study provided important insights into respiratory viral epidemiology in Bangladesh, underscoring the ongoing burden of multiple pathogens including HMPV [25]. It was the most common virus identified in febrile children, contributing to the high rates of respiratory illness in these populations [26]. Furthermore, the Institute of Epidemiology, Disease Control, and Research (IEDCR) in Bangladesh verified the presence of HMPV in a patient on January 12, 2025 [27]. The porous border between India and Bangladesh and frequent cross‐border mobility further amplifies this risk. Bangladesh, with its high population density, urban overcrowding, and limited healthcare resources, faces a substantial risk of the introduction and spread of HMPV. Despite these vulnerabilities, awareness of respiratory viral threats, including HMPV, remains low in Bangladesh. The rise in reported HMPV cases, such as in China and India, may partly reflect increased awareness and testing availability rather than a true epidemiological surge, as noted by World Health Organization (WHO) [28]. Therefore, strengthening general surveillance of respiratory infections is crucial. This perspective article emphasizes the urgent need for Bangladesh to proactively address the silent threat posed by HMPV. By establishing robust surveillance systems, enhancing healthcare capacity, and fostering regional collaboration, a country can mitigate the risk of HMPV outbreaks and strengthen its overall public health resilience.

## 2 Challenges in Addressing HMPV in Bangladesh

Tackling HMPV in Bangladesh is difficult due to a lack of proper diagnostic tools, poor reporting systems, and limited awareness among healthcare workers and the general public. As HMPV symptoms are very similar to those of other respiratory infections, it is difficult to diagnose and treat them quickly. The absence of a nationwide system to monitor HMPV cases and the shortage of advanced testing technologies makes it even more difficult to determine the spread of the virus. The situation is worse in rural areas, where healthcare services are harder to access, and many people, especially children and the elderly, have limited knowledge of health. These problems highlight the need for better monitoring systems, improved diagnostic tools, and awareness programs to effectively reduce the impact of HMPV.

## 3 Diagnostic Gaps

In Bangladesh, the challenges associated with diagnosing HMPV further complicate effective disease management. Conventional viral cultures and serological assays are often insufficient. VeroE6‐TMPRSS2 and Vero/TMPRSS2 cell lines, originally optimized for SARS‐CoV‐2 isolation, have also demonstrated high suitability for isolating HMPV [29, 30]. Incorporating such advanced methods can improve isolation capacity in Bangladesh. Molecular methods such as RT‐PCR have shown higher sensitivity, but remain underutilized owing to cost and resource constraints [31]. Innovative methods like CRISPR‐Cas12a‐based detection have emerged, providing rapid and sensitive diagnostics. Additionally, rapid antigen tests for HMPV are now available in several countries. Implementing rapid diagnostics can enhance early detection and help overcome infrastructural challenges in resource‐limited settings like Bangladesh. However, these technologies require additional validation for widespread clinical use in resource‐limited settings [32]. Multiplex RT‐PCR has been developed as a cost‐effective alternative, enabling simultaneous detection of HMPV and other respiratory viruses. This method could significantly reduce diagnostic costs in clinical settings in Bangladesh [18]. Although molecular assays such as real‐time RT‐PCR offer higher accuracy, their implementation is hindered by infrastructure limitations and the lack of trained personnel in Bangladesh [33]. Addressing diagnostic gaps for HMPV in Bangladesh requires adopting cost‐effective molecular diagnostic methods and strengthening laboratory infrastructure and training programs. Improved access to accurate diagnostics could enhance early detection and disease management for HMPV.

## 4 Lack of Surveillance

The lack of robust surveillance systems for HMPV in Bangladesh limits the understanding of its epidemiology, burden, and the implementation of effective public health interventions. Active surveillance in urban slums of Dhaka revealed HMPV as a leading cause of febrile respiratory illnesses in children. However, surveillance efforts remain isolated and insufficient to map the national burden [26]. HMPV is often excluded from routine diagnostic panels, leading to underreporting of cases. Studies indicate that including HMPV in routine multiplex PCR testing could improve its detection and epidemiological tracking [18]. Seasonal variations and co‐circulating genotypes complicate the surveillance of HMPV. For instance, studies have shown distinct genotypic variations within populations that are not consistently monitored in Bangladesh [34]. Unlike other respiratory pathogens, HMPV is not part of systematic respiratory disease surveillance programs in Bangladesh. Incorporating it can help identify outbreaks and guide resource allocation [35]. Emerging diagnostic tools, such as metagenomic sequencing and CRISPR‐Cas‐based assays, have demonstrated promise for rapid detection and could enhance surveillance efforts if incorporated into a national framework [32]. The lack of robust surveillance for HMPV in Bangladesh results in under‐detection and mismanagement of the virus. Strengthening surveillance by integrating HMPV into routine respiratory disease monitoring and adopting innovative diagnostic tools is critical for understanding its burden and improving public health responses.

## 5 Limited Public Awareness

Public awareness about HMPV is limited in Bangladesh, despite its significant contribution to respiratory illnesses. This lack of awareness hampers early diagnosis, prevention, and appropriate treatment of the disease. HMPV often presents symptoms similar to other respiratory infections, such as fever, cough, and shortness of breath, leading to misattribution to more well‐known diseases like influenza or pneumonia. This contributes to delays in seeking healthcare and receiving accurate diagnoses [26]. There are no widespread public health campaigns in Bangladesh focused on HMPV or respiratory viruses in general. Health education efforts targeting common symptoms and prevention strategies could improve early recognition and reduce transmission. Respiratory symptoms, particularly in densely populated and low‐income areas, are often stigmatized or normalized as common ailments. This normalization prevents individuals from understanding the potential severity of viral respiratory diseases, including HMPV [35]. Healthcare professionals often lack the resources or training to educate patients about less‐known viruses like HMPV. Increasing provider knowledge about HMPV could lead to better communication with patients about the risks, symptoms, and importance of prevention [33]. Vulnerable groups such as children, the elderly, and immunocompromised individuals are disproportionately affected by HMPV. Public awareness campaigns targeting caregivers and families could help prioritize healthcare access for these populations [36]. Limited public awareness of HMPV in Bangladesh hinders early diagnosis and prevention. National health campaigns and educational initiatives, supported by trained healthcare professionals, are crucial to bridge this awareness gap and reduce the burden of HMPV‐related respiratory illnesses.

## 6 Inadequate Research and Funding

Globally, HMPV research is underfunded and overshadowed by other respiratory viruses like influenza and RSV. In Bangladesh, the problem is exacerbated by limited healthcare budgets and competing health priorities, leaving significant gaps in understanding and addressing HMPV. Compared to other respiratory viruses, HMPV research receives less funding and attention globally. This lack of focus results in fewer studies, limited data, and inadequate resources for developing diagnostic and treatment protocols. Bangladesh lacks comprehensive studies on HMPV's epidemiology, seasonality, and impact. Most available data stems from isolated studies with limited scope, primarily conducted in urban settings like Dhaka [26]. With high burdens of diseases such as tuberculosis, dengue, and malnutrition, HMPV research is deprioritized in Bangladesh's healthcare agenda. Funding for respiratory viruses is often directed toward pathogens with higher perceived urgency, such as influenza. Limited research funding hinders the development of cost‐effective and locally adapted diagnostic tools for HMPV, restricting its detection and surveillance [31]. Inadequate research and funding for HMPV in Bangladesh leave critical gaps in understanding the virus's epidemiology and impact. Prioritizing research investment and leveraging international partnerships can help bridge these gaps and improve public health responses to HMPV.

## 7 Policy Recommendations

The development of a National Respiratory Pathogen Surveillance Program is essential for strengthening public health infrastructure against respiratory infections. Surveillance should not be limited to a single virus but must encompass multiple pathogens, including HMPV, influenza, and RSV. High‐quality surveillance should prioritize standardized case definitions, fixed diagnostic protocols, and quality over quantity of testing. Even small‐scale sentinel surveillance with high diagnostic accuracy can provide valuable epidemiological insights. A robust and integrated surveillance system encompassing data collection from hospitals, clinics, and laboratories will enable real‐time monitoring of viral circulation. This capability is pivotal for the early detection of outbreaks, timely response measures, and reduction of the disease burden.

Collaborative partnerships with national health authorities and international organizations, particularly the WHO, are crucial for the success of such programs. Real‐time data sharing and coordinated efforts will enhance regional preparedness and facilitate a harmonized response to emerging and re‐emerging respiratory pathogens. These partnerships should prioritize the establishment of standardized protocols for case identification, data reporting, and outbreak management to ensure consistency and reliability across surveillance networks.

Capacity building within health care systems is critical. Structured training programs should be implemented to enhance healthcare workers' competencies in recognizing, diagnosing, and managing HMPV infection. Furthermore, expanding the diagnostic capabilities by equipping healthcare facilities with advanced tools and technologies will enable accurate and timely pathogen identification. This infrastructural enhancement is indispensable in mitigating the impact of respiratory infections.

Public engagement through evidence‐based awareness campaigns is another cornerstone for effective disease management. These campaigns should emphasize key preventive measures such as respiratory hygiene practices, vaccination advocacy, and the importance of seeking early medical interventions for respiratory symptoms. Utilizing a multichannel communication strategy, including digital platforms and collaboration with community leaders, can maximize the reach and efficacy of these initiatives.

Finally, promoting research on HMPV and related respiratory pathogens is critical for advancing the scientific understanding of their epidemiology, transmission dynamics, and clinical impact. The research findings should inform public health policies and intervention strategies, ensuring that responses are grounded in robust evidence. Interdisciplinary research collaborations supported by adequate funding are essential for addressing the complexities of respiratory pathogen management.

## Author Contributions

**Md Jubayer Hossain:** conceptualization, investigation, writing – original draft, writing – review and editing, validation, project administration, resources, supervision, data curation, formal analysis. **Soniya Akter Sony:** writing – original draft, writing – review and editing. **Fatema Tuj Johora Fariha:** writing – original draft, writing – review and editing. **Sajjad Hossen:** writing – original draft, writing – review and editing.

## Conflicts of Interest

The authors declare no conflicts of interest.

## Transparency Statement

The lead author Md. Jubayer Hossain affirms that this manuscript is an honest, accurate, and transparent account of the study being reported; that no important aspects of the study have been omitted; and that any discrepancies from the study as planned (and, if relevant, registered) have been explained.

## Data Availability

The authors have nothing to report.
